# Cubitus Valgus with Tardy Ulnar Nerve Palsy - Is Anterior Transposition of the Ulnar Nerve Always Necessary? A Case Report

**DOI:** 10.5704/MOJ.2007.010

**Published:** 2020-07

**Authors:** IM Anuar-Ramdhan, R Remli, AH Abdul-Rashid, S Ibrahim

**Affiliations:** 1 Department of Orthopaedics, Sarawak General Hospital, Kuching, Malaysia; 2 Department of Medicine, Universiti Kebangsaan Malaysia Medical Centre, Kuala Lumpur, Malaysia; 3 Department of Orthopaedics and Traumatology, Universiti Kebangsaan Malaysia Medical Centre, Kuala Lumpur, Malaysia

**Keywords:** anterior transposition, cubitus valgus, ulnar neuropathy

## Abstract

Tardy ulnar nerve palsy is a known complication of cubitus valgus. The options for treating the ulnar neuropathy include anterior nerve transposition or neurolysis. We report on an 11-year-old boy who had a tardy ulnar nerve palsy due to cubitus valgus resulting from a non-union of a lateral condyle fracture of the humerus. Anterior transposition of the ulnar nerve was not done after the closing wedge osteotomy of the distal humerus. The close wedge osteotomy relieved the tension on the nerve and not transposing the ulnar nerve anteriorly prevented an iatrogenic nerve injury. The patient had no restriction with activities of daily living at the six years follow-up although neurological recovery was incomplete.

## Introduction

Lateral humeral condyle fractures are relatively common in children. As it is intra-articular, immediate intervention is required, especially in displaced fractures, to restore the anatomical congruity of the elbow joint.

The common elbow deformity following an untreated lateral humeral condyle fracture is cubitus valgus. It is a sequela of non-union or malunion of the lateral humeral condyle. Patients with post-traumatic cubitus valgus may present in several ways including limitation of elbow motion, pain, joint instability as well as tardy ulnar nerve palsy.

Anterior transposition of the ulnar nerve in cases of tardy ulnar nerve palsy has been recommended by most surgeons^1, 2, 3^. We report a case of cubitus valgus with ulnar neuropathy that had been treated with corrective osteotomy of the distal humerus without transposing the ulnar nerve anteriorly.

## Case Report

An 11-year-old boy presented with left elbow deformity and clawing of the left little and ring fingers with numbness over the medial aspect of the hand. He injured his left elbow after falling from a tree at the age of six years. He was treated initially with a traditional healer. He started to develop progressive left elbow deformity with clawing of the little and ring fingers and hypoesthesia over the ulnar aspect of the left hand one year after the injury.

**Fig. 1:**
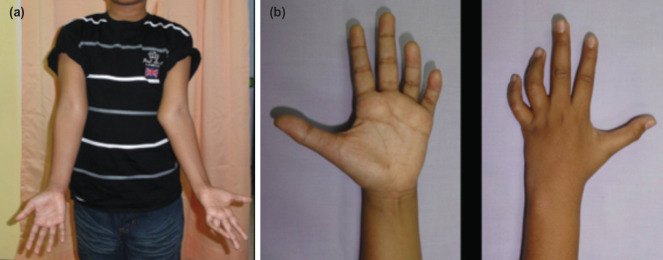
Pre-operative appearance of the left elbow and left hand. (a) Left cubitus valgus with prominent medial epicondyle. (b) Left ulnar claw hand with wasting of the intrinsic muscles.

**Fig. 2:**
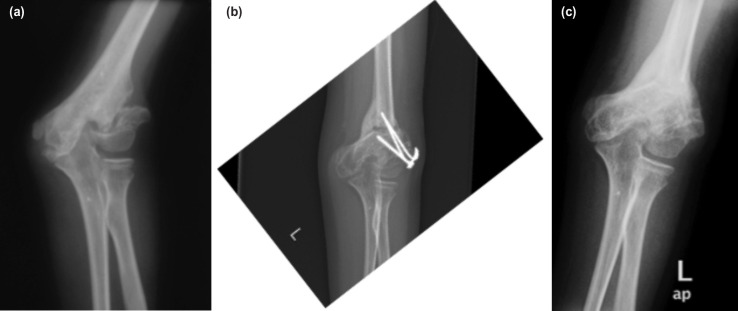
Radiograph of the left elbow. (a) Pre-operative film showing left cubitus valgus with non-union lateral humeral condyle. (b) Post-operative film after corrective osteotomy in the process of healing two months after surgery. (c) Post-removal of implants after three months.

Physical examination revealed a left cubitus valgus with an ulna claw hand, with weakness and wasting of the intrinsic muscles of the hand (Fig. 1). The sensation was reduced over the ulna distribution. The Froment test was positive. The radiograph showed non-union of the lateral condyle of the left humerus (Fig. 2a).

The diagnosis was a left cubitus valgus secondary to the non-union of the lateral condyle of the humerus with tardy ulna nerve palsy.

The patient was treated by fixing the lateral condyle in-situ with a single cancellous screw after excising the soft tissue and freshening the bone edges of the non-union. Bone grafting was not used. From the same lateral approach, the cubitus valgus was corrected by a supracondylar medial closing wedge osteotomy. The osteotomy level was determined and performed at the centre of rotation and angulation (CORA) and stabilised with two Kirschner wires (Fig. 2b). A 30° bone wedge was removed. The ulnar nerve was not transposed anteriorly. A plaster slab was applied for four weeks. The implants were removed after bone healing at three months (Fig. 2c).

Recovery of ulna sensation was noted six weeks after surgery. The clawing of the ring and little fingers resolved one year after surgery.

**Fig. 3:**
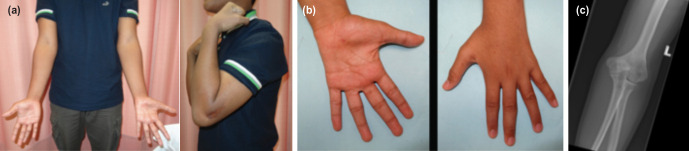
Clinical and radiological photographs four years after surgery. (a) Left elbow deformity corrected with full range of elbow movement. (b) Resolution of the claw hand deformity. (c) Radiograph showing complete healing with a fish-tail deformity of the distal humerus.

Four years after surgery, the left elbow had full range of motion with no deformity (Fig. 3a). The claw hand deformity had resolved, but there was residual hypoesthesia over the ulnar distribution and weakness of the intrinsic muscles (Fig. 3b). Further remodelling of the distal humerus had occurred (Fig. 3c)

A nerve conduction study of the left upper limb was done five years after surgery. The study showed an absent ulnar motor compound muscle action potential with the absence of both ulnar and dorsal ulnar cutaneous sensory nerve action potentials consistent with left ulnar neuropathy at the elbow region. Despite the absent motor and sensory potentials of the left ulnar nerve, the needle electromyography of the left first dorsal interosseous and left abductor digiti minimi muscles showed giant polyphasic motor unit action potentials consistent with chronic re-innervation changes suggestive of previous nerve injury.

The latest follow-up was six years after surgery when the patient was 17-years-old. There was no further improvement in the neurological recovery of the left hand. However, the patient had no restriction in activities of daily living.

## Discussion

The ulnar nerve is relatively fixed behind the medial epicondyle of the humerus. Any increase in a valgus deformity at the elbow joint would lead to stretching of the nerve resulting in neuropraxia. This condition is temporary and usually recovers with remyelination of the axon that takes about three weeks to three months^4^.

If the deforming force is progressive, it might injure the axon within the endoneural tube of the nerve with intra-neural scarring and fibrosis resulting in axonotmesis. The outcome of axonometsis is variable, depending on the magnitude of the intra-neural injury^4^. Neurotmesis is uncommon in tardy ulnar nerve palsy.

The recovery of the ulnar nerve will depend on the severity of the nerve injury pre-operatively and the correction of the deformity after surgery. Most authors recommend anterior transposition of the ulnar nerve to release the tension. This can be done with or without correcting the cubitus valgus deformity^1, 2, 3^. Mortazavi *et al* found that not all patients in his series showed improvement of ulnar nerve symptoms after the anterior nerve transposition^2^. Kang *et al* reported similar findings as the recovery of the nerve differed even after anterior transposition^1^.

We treated our patient without transposing the ulnar nerve anteriorly as we believed that correcting the valgus deformity with a closing-wedge osteotomy would relieve the tension on the ulnar nerve. Furthermore, the anterior transposition of the ulnar nerve could lead to an iatrogenic injury of the nerve^5^. We have shown that nerve recovery in our patient did occur, but it was incomplete six years after surgery. The injury was most probably an axonotmesis, and long-standing nerve stretching before surgery contributed to the outcome of the nerve recovery.

The nerve conduction and electromyography studies five years after the surgery was consistent with a chronic re-innervation process of an isolated left ulnar neuropathy at the elbow with partial recovery. However, there was no further clinical improvement in neurological recovery at the six years follow-up.

In conclusion, the correction of the cubitus valgus deformity at the CORA with 30° medial closing wedge osteotomy released the tension on the tethered ulnar nerve without requiring an anterior transposition of the nerve. The 30° correction also allowed recovery of the nerve with no restriction in the hand function.
